# Abdominal Plain Radiograph in Neonatal Intestinal Obstruction

**DOI:** 10.21699/jns.v6i1.483

**Published:** 2017-01-01

**Authors:** G Raghavendra Prasad, Amtul Aziz

**Affiliations:** Deccan College of Medical Sciences and Princess Esra Hospital, Hyderabad

**Keywords:** Abdominal radiographs, Intestinal obstruction, Neonate

## Abstract

A comprehensive all-inclusive resource on plain radiograph in neonatal intestinal obstruction is presented. This is an attempt to develop a protocol and to regain expertise in evaluating a plain radiograph that most often yields more than enough clues to diagnose and to decide a plan of action.

**Introduction:**

Plain radiograph of abdomen was and continues to be a great and useful tool and diagnostic array of neonatal intestinal obstruction. It was the only tool in the past. Irrational use of ultrasound, inadequate training of radiologist with regards to plain radiograph, advance imaging modalities like CECT have blurred the importance of plain radiograph of abdomen. A bedside analysis of plain radiograph of abdomen is slowly disappearing. We attempt to revisit and create a resource for pediatric surgeons, pediatricians, and radiologists alike. We aim to describe the methodical approach to analyze plain radiograph of abdomen.

Neonatal intestinal obstruction is conventionally defined as intestinal obstruction from duodenum to anal canal; esophageal and gastric outlet obstructions are conventionally excluded. The first and foremost important investigation to evaluate neonate with intestinal obstruction is a plain radiograph of abdomen.

Ideal views that need to be taken include: Supine film, Erect film, Decubitus film, Cross-table lateral view, Right side up left side up decubitus film, Lateral view, and Films after injecting air.

If only all views are adopted in standard protocol of bedside imaging, evaluation would be comprehensive and complete and unnecessary repetition can be avoided. All views as standard imaging protocol is recommended. Look for these features systematically while evaluating the plain radiograph: a) Whether complete abdomen is included or not? b) Is the abdomen distended/scaphoid on radiograph? c) What is the gas pattern? d) Is the gas pattern symmetric/asymmetric? e) Any localized area of haziness, f) Any area of honeycomb or gas in gut wall, g) Any gas seen in scrotum, h) Type and number of fluid levels, i) Any extra intestinal gas/air, j) Liver shadow, k) Any other mass seen, l) Any gas seen in bladder area, m) The vertebrae seen and their pattern, n) Lung seen in lower part, o) Pleural spaces, p) Diaphragm on either side, q) Heart and mediastinum, r) Any calcification seen, s) Description of indwelling catheters tubes and surface electrodes.

Eye catching misleading shadows are; normal shadow of cord, soft tissue produced by penis, and probes, electrodes, transducers etc.

Newborn swallows air from the very first cry after birth. Gas is usually seen in entire small bowel by 3hours; it reaches the rectum by 12 hours. Normal gastric bubble is usually under the left dome of diaphragm; both greater and lesser curvatures can be made out. A horizontal fluid level can be normally seen if child is fed recently. Normal intestinal gas pattern is honeycomb shaped, almost symmetrical polygonal gas/air spaces with thin walls. The intestinal loops are defined as dilated or and distended if diameter of loop is more than the transverse diameter of vertebrae or as big as surgeons thumb, the so-called Thumb sign.

A No.8 nasogastric tube/ or No.10 oro-gastric tube should be mandatorily put in all such surgical neonates. In a neonate, gastro-intestinal obstruction (mechanical or functional) would produce abnormal air patterns and air-fluid levels that would differ as per the different sites of gastro-intestinal obstruction. [1,2]

Large and transverse solitary fluid level in left upper abdomen, often crossing the midline, is a gastric fluid level, or gastric bubble. It indicates gastric outlet obstruction. Other abnormalities to look for about gastric bubble include the following; Abnormally high placed gastric bubble is seen in, i) eventration dome of diaphragm or left sided phrenic palsy (secondary to birth injury), ii) diaphragmatic hernia with sac, iii) hiatus hernia.

Upside down/mirror image stomach indicates gastric volvulus.

Absent gastric bubble indicates pure esophageal atresia; gasless abdomen in such a case can obscure the signs that we expect to see in distal gut obstructions, e.g., duodenal atresia.

A double bubble without any gas below is diagnostic of duodenal atresia; the gastric bubble here may be very large (Fig.1). If there is double bubble with paucity of gas distally, then think of malrotation with volvulus, unless proved otherwise. A duodenal diaphragm depending on its site will also present with a gastric bubble or double bubble, but there would be significant gas distally. Duodenal fluid level is usually on midpoint of right side of abdomen; it does not cross midline (Fig.2).

**Figure:**
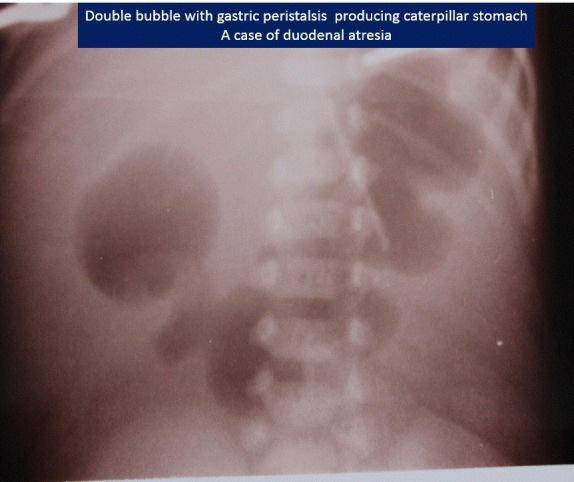
Figure 1: Duodenal atresia with gastric caterpillar sign.

**Figure:**
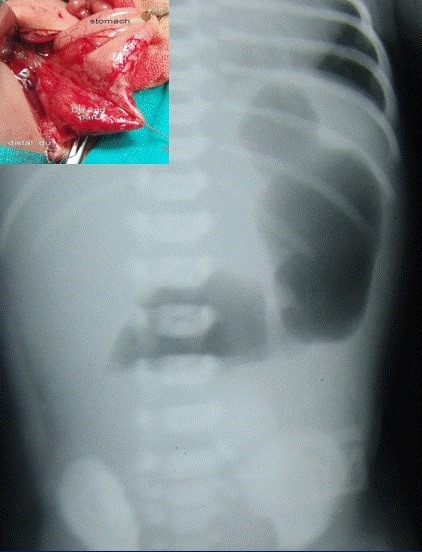
Figure 2: Double bubble sign. Inset shows duodenal atresia.

**Figure:**
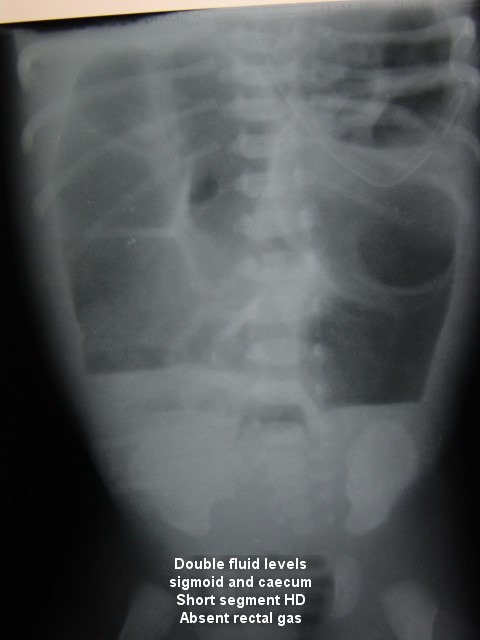
Figure 3: X-ray showing absent gas in the pelvis- aganglionosis of distal colon.

**Figure:**
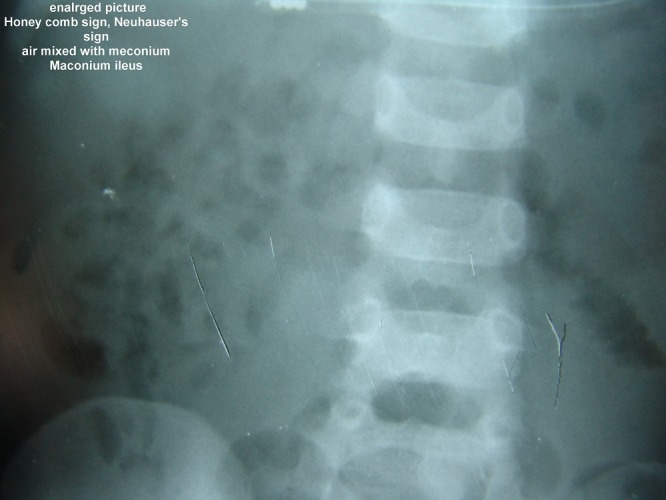
Figure 4: Neuhauser’s sign in meconium ileus.

Jejunal fluid levels, usually only few numbers, in left upper abdomen are seen with proximal jejunal atresia. Classical spring coil effect produced by mucosa of jejunum is usually not prominent in neonatal period. [3]

Distal jejunal or ileal obstruction would result in multiple, asymmetric centrally placed fluid levels that often of different sizes; the biggest is just proximal to the site of obstruction. Stepladder pattern may be seen. The differential diagnoses would include the differential diagnosis being ileal atresia, meconium ileus, ileal stenosis, caecal atresia (proximal), total colonic aganglionosis, obstructed inguinal hernia, rare internal hernia.

Caecum and sigmoid colon fluid levels along multiple air fluid levels of small bowel will indicate distal anorectal obstruction as seen in classical Hirschsprung’s disease or rectal atresia (Fig.3).

Large air fluid levels crossing midline with small bowel loops pushed to the side indicates pouch colon syndrome. (Fig.13)

Honeycomb air trapping with paucity fluid levels – Neuhauser’s sign. (Fig.4)

Other patterns of fluid levels include: Fluid levels lateral to right border of liver indicate subphrenic abscess, pneumoperitoneum; Fluid levels under diaphragm indicates subphrenic abscess with intestinal source or perforation; Isolated fluid level in radiograph like fluid level in bladder indicate recto-urinary communication.

**How to differentiate large bowel from small bowel on plain radiograph?**

Small bowel cannot be differentiated from large bowel on plain radiograph. This is due to non-development of haustrae. This can be accomplished by barium enema.

**Role of lateral radiograph:**

Lateral radiograph helps to differentiate abnormal gas filled/fluid levels containing structures such as: Stomach on lateral radiograph anterior transverse fluid level under left dome of diaphragm; Duodenum is seen near colon, posterior; Air in the bladder is seen in anterior retropubic region; Portal vein gas shadow which is centripetally placed seen in advance NEC.

**Prone cross table lateral view:**

It helps to differentiate mechanical from functional obstruction for ileum. It also serves an alternative to invertogram in ARMs. Absent air indicates mechanical obstruction. Rectal gas suggests ileus.

**Decubitus films:**

It helps to pick small pockets of extra intestinal air/ gas these films shows the free air in non-dependable parts of abdomen.

**Air contrast studies:**

Could be done for better differentiation in some conditions;

Upper GI air contrast studies are valuable in the diagnosis of duodenal obstruction. If 20-30ml of air is injected into the stomach while the neonate is in right side up lateral position, it will be trapped in proximal dilated duodenum. It would help to differentiate duodenal atresia from malrotation. Beak sign / spring coil sign seen in malrotation with midgut volvulus could be better appreciated this way. At times in neonates with congenital diaphragmatic hernia, this study may be necessitated to confirm the stomach either in the chest or in the abdomen.

Lower GI air contrast studies would help to know the distal most site of obstruction. Air-filled recto-sigmoid might suggest Hirschsprung’s disease. A lateral view may even delineate if the intermediate zone in a neonate with Hirschsprung’s disease lies in recto-sigmoid region.

Air as contrast in these GI studies has the following advantages: Available free globally; Nontoxic; Does not require special equipment; Incidence of complications or iatrogenic injuries is low, and even if it happens, it does not lead to chemical peritonitis.

There are other features beyond abnormal gas intestinal patterns and air-fluid levels that need to systemically looked for in an abdominal radiograph.

**Abnormal gas pattern(s): **(5)

No description of plain radiograph is complete without noting details of presence and absence of extra-intestinal gas.

Air under dome of diaphragm or in various compartments of abdomen (6,7); Air in entire abdomen will produce football sign (Fig.5); Air under both domes of diaphragm will produce saddle bag sign (Fig.5); Air under central tendon produces copula sign; Air in peritoneal cavity with air in falciform ligament (Fig.6); Air in free layers of falciform ligament pushing layers to one side will produce linear sign (Fig.6); Air between loops of bowel with air inside the intestine will produce silver lining or Rigler sign showing serosa of intestine; Triangular sign is due to trapped free air between two loops of intestine and the intestinal loops and abdominal wall; Free air can also show and delineate shape of solid organs like liver kidney and spleen; Visualization of right border of liver indicates pneumoperitoneum; Ligaments sign: urachus, umbilical arteries may be seen as linear, vertical/ oblique lines respectively; Retroperitoneal free air can dissect an iliopsoas and will produce oblique linear sign; Free air dissecting mesentery and rectum can ascend in mediastinum will produce pneumomediastinum; Free gas in scrotum is seen in NNEC, obstructed gangrenous inguinal hernia; Free air dissecting other ligaments of body can show up the ligaments on abdomen.

**Figure:**
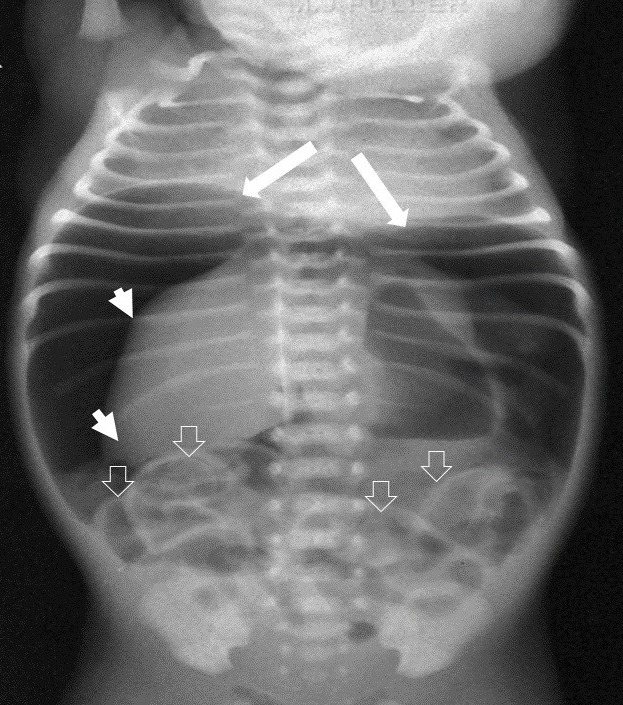
Figure 5: Pneumoperitoneum. Big white arrows showed air under diaphragm leading to its elevation. Small white arrows delineate pushed down liver border. Empty arrows show Rigler’s sign.

**Figure:**
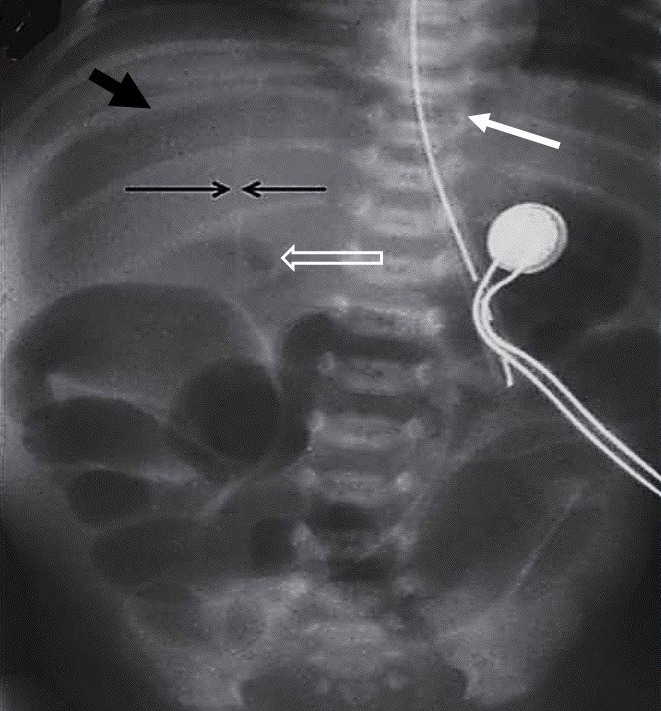
Figure 6: A case of gastric perforation showing pneumoperitoneum. Thick black arrow showed gas in front of liver making it lucent. Thin small arrows captured ligamentum teres. White arrow showed gas under central tendon (cupola sign). Hollow white arrow showed gas in Morrison’s pouch.

**Figure:**
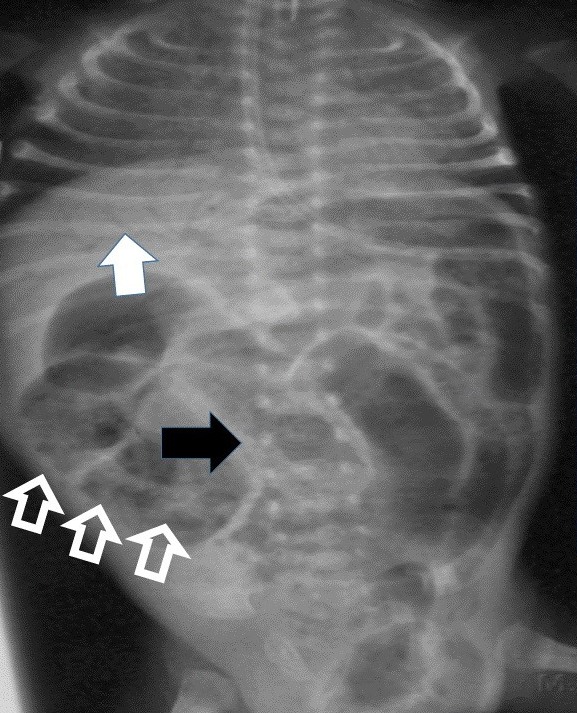
Figure7: Pneumatosis intestinalis in a patient of NEC. White arrow showed portal venous gas. Black arrow linear or string sign. Hollow white arrow showed soap bubble sign.

**Other abnormal signs/sites:**

Air gas in gut wall is called pneumotosis cystoides intestinalis (Fig.7) [8]; Gas in the gut wall is a sign of intestinal obstruction with ischemia characteristically seen in necrotizing enterocolitis of newborn.

Clustering of loops in one area with the rest of the abdomen being gasless could be seen in following conditions (Fig.8,9): Mass lesions pushing the loops to one side; Loculated pneumoperitoneum, cystic meconium peritonitis; Congenital abdominal cocoon; Internal hernias.

Free gas in portal vein is seen in newborn in necrotizing enterocolitis of newborn and or after biliary-enteric anastomosis.

Gas delineating psoas shadow

Perirenal air nephrogram

Scrotal gas

**Figure:**
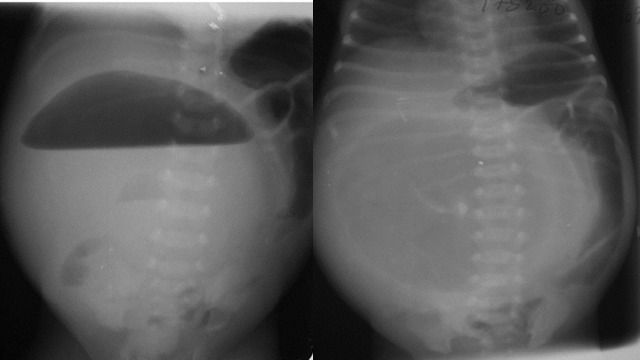
Figure 8: Giant cystic meconium peritonitis.

**Figure:**
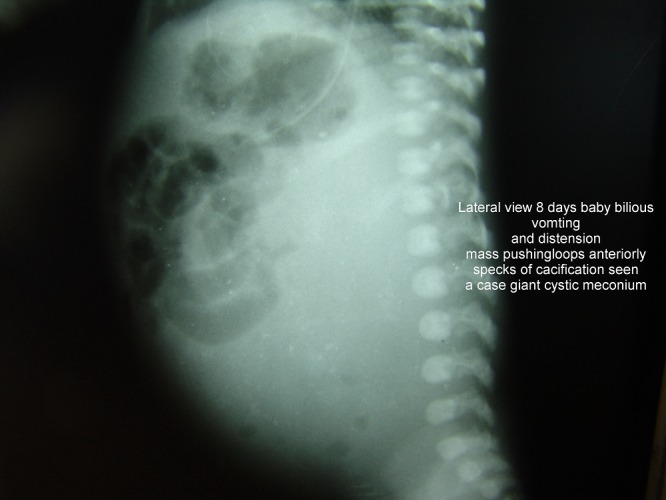
Figure 9: lateral view in giant meconium cyst. Note calcifications.

**Figure:**
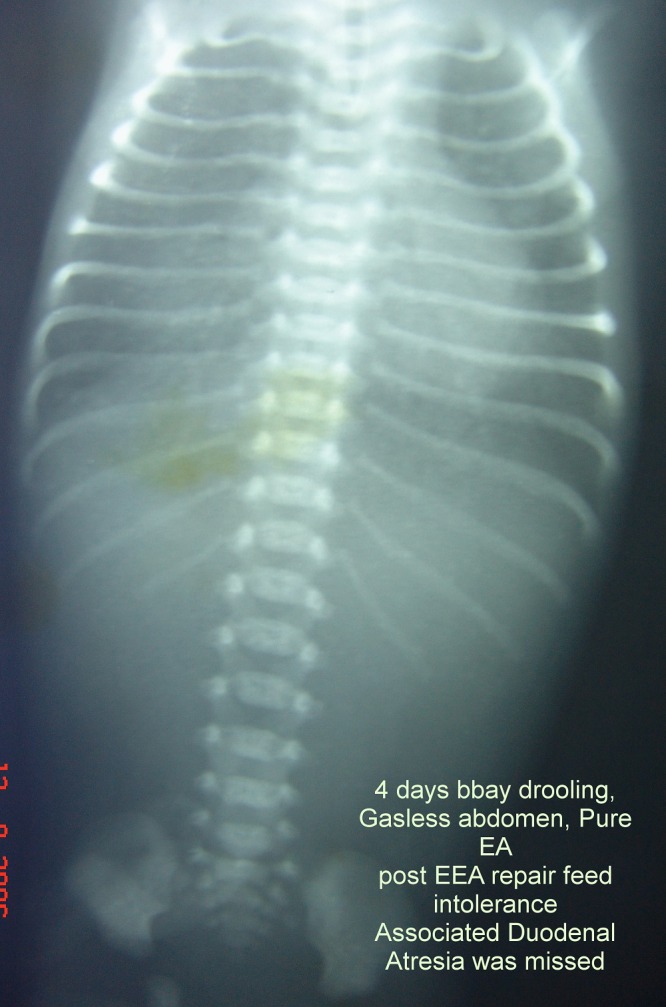
Figure 10: Gasless abdomen.

**Figure:**
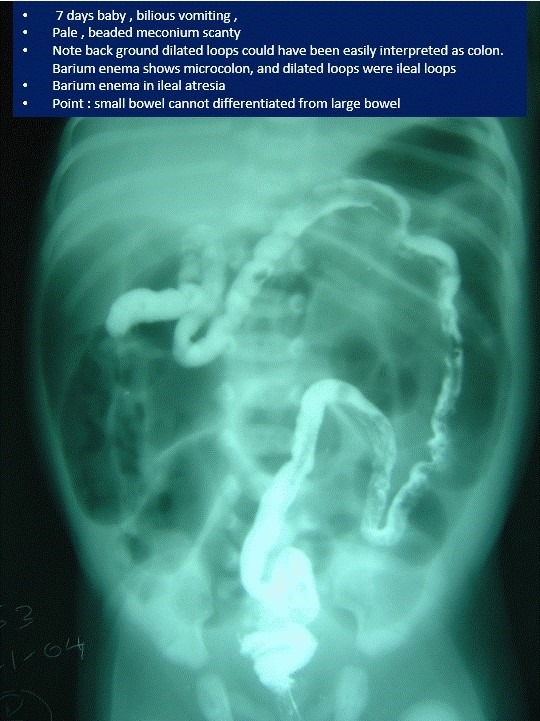
Figure 11: Barium enema showing microcolon in a case of ileal atresia.

**Figure:**
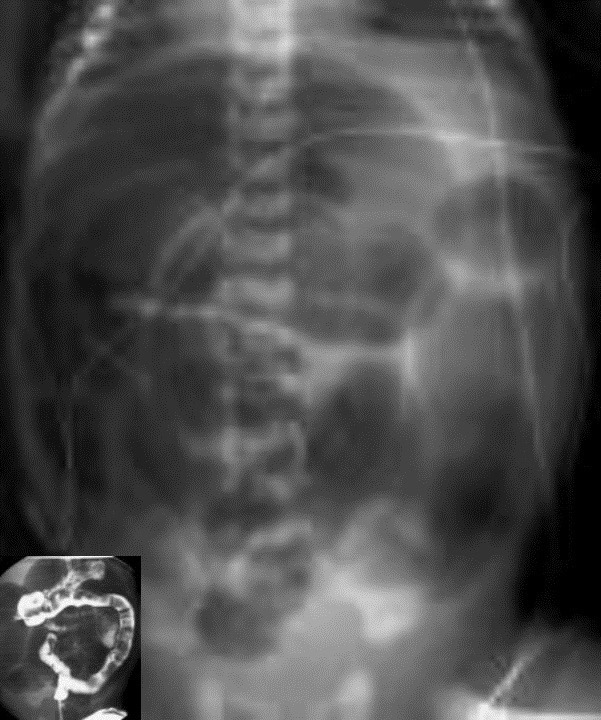
Figure 12: X-ray showing dilated bowel loops. Contrast enema showed filling defects seen in meconium plug syndrome.

**Gas less abdomen:** (Fig.10)

It usually indicates intra-abdominal catastrophe; causes include: Persistent nasogastric aspiration; Massive gangrene of small bowel; Massive ascites; Massive intra-abdominal tumors; Giant cystic meconium peritonitis, massive duplication cyst and omental cyst (Fig.8,9).

Collateral signs include: Calcification; Abnormal pockets of gas; Abnormal vertebrae; Skeletal anomalies.

Intra-abdominal calcifications could be seen in the following: Meconium ileus and meconium peritonitis; Intra-abdominal tumors like neuroblastoma, hepatoblastoma and rarely Wilms’ tumor, retroperitoneal terato-dermoids; Calcification of hemorrhagic adrenal cyst will produce ring sign.

Beware of the misleading shadows:

1. Umbilical stump with clip might be confused with fetus in fetu.

2. Penis can show a coin-shaped shadow that can be mistaken or pathological lesion.

3. Nasogastric tube: linear shadow through entire part of chest going into upper abdomen. Rarely excessive length can be extended into duodenum.

4. Umbilical venous catheter: upwards to the right might go into IV.

5. Umbilical arterial catheter: downwards laterally.

6. Esophageal transducer: long linear tubes with distal transducer in mid/lower esophagus.

7. Rectal transducers: usually seen pelvic area around 4-8cm.

8. ECG electrodes: chest.

9. Skin temperature electrodes: larger shadow thicker

Some more misleading shadows: Beware of bulb of Foley’s catheter; Beware of straps of pampers; Beware of foreign bodies left on mattress of baby like coins, intra venous catheters, pens mobiles of staff.

Barium enema-An extended and essential supplement part of plain radiograph

First enema child should receive should be barium enema.

It should be done before rectal examination and before stimulating rectum with tube or thermometer.

Barium enema helps to see: big colon; normal colon; microcolon; discontinuous colon; and duplicate colon.

Inability to perform Barium enema with intact normal anus might suggest rectal atresia.

How to differentiate microcolon due to total colonic aganglionosis from other differential diagnoses such as ileal atresia, left small colon syndrome, (Fig.11,12) meconium ileus, rarer causes of caecal/colonic atresia, meconium plug syndrome, on barium enema?

1. Total colonic aganglionosis shows a small-unused colon.

2. Loss of hepatic and splenic flexures. This gives rise to “question mark- ?” colon.

3. Small colon or microcolon with multiple filling defects are seen in meconium plug syndrome.

4. Small colon with free reflux of barium into distal ileum is seen in ileal atresia.

5. Small colon with refluxed barium showing multiple filling defects is seen in meconium ileus.

**Figure:**
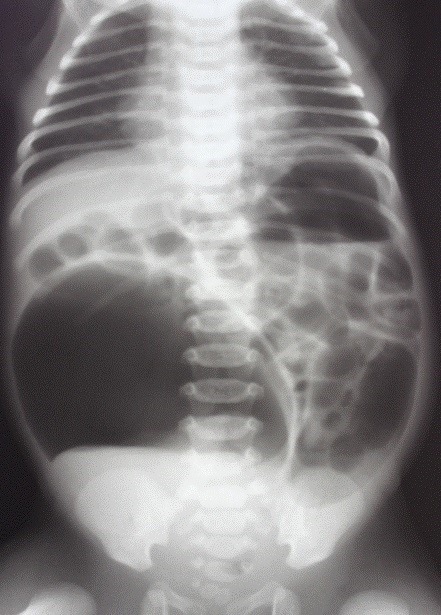
Figure13: Big gas shadow of congenital pouch colon pushing gut loops to left abdomen.

## Footnotes

**Source of Support:** Nil

**Conflict of Interest:** None
